# Organizational Commitment and Intention to Leave of Nurses in Portuguese Hospitals

**DOI:** 10.3390/ijerph19042470

**Published:** 2022-02-21

**Authors:** Teresa Neves, Pedro Parreira, Vitor Rodrigues, João Graveto

**Affiliations:** 1 Health Sciences Research Unit: Nursing (UICISA: E), Nursing School of Coimbra (ESEnfC), 3000-232 Coimbra, Portugal; parreira@esenfc.pt; 2 Faculty of Medicine, University of Coimbra, 3000-548 Coimbra, Portugal; vrodrigues@fmed.uc.pt

**Keywords:** intention to leave, organizational commitment, nursing staff, work environment, hospitals

## Abstract

Intention to leave is influenced by the commitment and individual and structural factors. It is a critical dimension in health systems due to the shortage of professionals and the potential impact on the quality of care. The present paper: (i) characterizes organizational commitment and intention to leave; (ii) analyzes the relationship between structural factors (such as, work environment and nurse staffing), individual factors (age), and nurses’ organizational commitments and intention to leave; and (iii) analyzes the differences in the intention to leave and in the organizational commitment according to service specialty, nurses’ specialization, and contractual relationship in Portuguese public hospitals. A cross-sectional study was conducted with a sample of 850 nurses from 12 public hospitals units. The results show a high affective and continuance commitment of nurses with the hospital, and a reduced tendency of the intention to leave. A significant positive association was also found between the intent to leave and individual/structural factors. Organizational commitment and intention to leave levels are satisfactory, despite the influence of several factors, such as nurse staffing, work environment, or other opportunities for professional development. The results identify particularly sensitive areas that, through adequate health and management policies, can reduce nurses’ intentions to leave and promote the sustainability of the health system.

## 1. Introduction

Nursing, the largest professional group in health systems, plays a key role to ensure the quality of care and patient safety. The shortage of nurses is a current, serious social issue. Current trends indicate a global shortage of 5.7 million nurses by 2030 [1]. The increase in health needs, resulting from sociodemographic and epidemiological changes, tend to aggravate this problem by increasing the need for nurses. However, the supply of nurses is not expected to keep up with these growing needs. Furthermore, the turnover and intention of professionals to leave exacerbates this problem. These are influenced by organizational and individual factors, such as organizational commitment, hostile environments, and poor work conditions, which affect the nurses’ retention and quality of care [1,2,3,4,5,6].

Within this context, increasing attention has been focused on turnover and intention to leave, due to their obvious influence on organizational performance, resulting in financial and human resource losses. This is particularly relevant in healthcare institutions and for nurses, due to the complexity of care settings and the effect on performance and quality of care [5,7,8,9].

Intention to leave can be described as the desire to quit and seek a new unit/department within the same institution (internal) or in another institution (external), and may also include leaving the profession altogether. This is a complex phenomenon, especially in healthcare settings, and can be influenced by multiple factors, including motivational, cognitive, and behavioral factors [10,11].

According to previous studies, factors that include work environment, emotional exhaustion, practice safety, and shortage of staff, associated with professionals’ characteristics (such as age and education), can bring about nurses’ desire to leave their unit, department, or organization. Inadequate staffing, including an increased workload in work environments unfavorable to nursing practices, usually motivates professionals to leave [4,7,12,13]. Professional development opportunities are also a determining factor. The lack of career advancement has been identified as a predictor of organizational commitment and intention to leave [11].

Furthermore, organizational commitment has been assumed as one of the predictors of intention to leave [14]. Organizational commitment is defined as the employee’s psychological attachment to the organization. According to Meyer and Allen [15,16], it can be characterized through a three-dimensional approach with the following components: affective organizational commitment, defined by the emotional attachment by which employees identify themselves with the organization’s goals and values; continuance organizational commitment, associated with the cost of leaving the organization, implied by personal investment and expected profit; and normative organizational commitment, in the case of feelings of moral obligation to stay with the organization, which indicates little motivational involvement [15,16,17].

Organizational commitment is influenced by personal and structural factors and the employee’s previous experience. The continuance organizational commitment is particularly relevant in the healthcare context and should be promoted to mitigate a nurse’s intention to leave. It is the older nurses who tend to show more continuance organizational commitment and less intention to leave [18]. The most committed professionals manifest less intention to leave, in an inverse relationship between these two variables. Individual factors, such as age and education, also correlate significantly with intention to leave and organizational commitment [19]. Professionals’ characteristics and the organization’s structure affect the organizational commitment. In the professional category of nursing, commitment is identified as predictive of intention to leave and turnover, also determining absenteeism [20]. 

In Portugal, the concern with retaining nurses is essential, given that it maintains ratios of nurses per capita clearly below the OECD average [21]. The global economic and financial crisis of 2007–2008 led to the formulation of cost control measures in Portugal, including reducing salaries, stopping career progressions and promotions, and increased work hours, affecting work conditions [22]. There was also a reduction of 3.2% in the nursing staff in the National Health Service (NHS) between 2011 and 2013. At the same time, there was an exponential increase in the emigration of Portuguese nurses [23].

Taking into account that nurse turnover is a costly phenomenon for healthcare institutions that jeopardizes the quality of care and induces financial waste [9], given the Portuguese context, it is crucial to analyze this phenomenon. This is essential to underpin decision-making and health and human resource management policies.

However, in Portugal, studies on these matters are scarce. A previous study carried out in the Portuguese context reported the influence of individual and structural variables on the intention to leave of nurses [24]. However, it did not analyze factors, such as the contractual relationships, or the context of care delivery in the specialty dimension.

In this regard, the present study aims: (i) to characterize organizational commitment and intention to leave; (ii) to analyze the relationship between structural factors (such as work environment and nurse staffing), individual factors (age), and nurses’ organizational commitment and intention to leave; and (iii) to analyze the differences in the intention to leave and in the organizational commitment according to specialty, professional title of the specialist nurse, and the contractual relationship of nurses in Portuguese public hospitals.

## 2. Materials and Methods

### 2.1. Study Design, Participants, and Setting

In the present study, an observational, cross-sectional study was carried out. This followed the Strengthening the Reporting of Observational Studies in Epidemiology (STROBE) reporting guidelines [25].

The target population included all the nurses from 79 inpatient services of general surgery, internal medicine, and orthopedic specialties from 12 public hospital units. Groups I, II, and III hospitals (classification according to the Portuguese Ordinance No. 82/2014 (Portaria No. 82/2014)) were included. These represent different levels of response complexity, differentiation of care, hierarchization, and the proximity of healthcare services, in the coastal and inland, central, and northern regions of Portugal.

Given the objectives of the study, it was decided to include in the sample all the elements of the target population who voluntarily agreed to participate in the study (convenience sampling). A total of 1844 nurses were invited to participate. The inclusion criterion was working in direct care delivery, excluding nurses with management functions. 

### 2.2. Variables, Instruments, Data Collection, and Procedures

The data collection took place between January and September 2015. 

The data collection instrument contained an introduction letter, presenting the topic, scope, and purpose of the study, as well as a self-reported questionnaire, and a free informed consent form to sign. To avoid bias, participants answered the questions without the researcher being present. The nurse managers mediated the distribution and collection of the instruments in the services. The participants completed the questionnaire according to their availability. The questionnaire and consent form were collected in two separate (individual) envelopes to ensure total anonymity. All data collection instruments were alphanumerically encoded to preserve the participants’ identities.

To achieve the study objectives, the instrument included (i) sociodemographic questions (age and gender) and questions to characterize the professional situation, (ii) the Intention to Leave Scale, (iii) the Portuguese version of the Organizational Commitment Questionnaire, and (iv) the Portuguese version of the Practice Environment Scale of the Nursing Work Index (as a structure factor). 

The Intention to Leave Scale, adapted by Mendes [26], is a scale for evaluating intention to leave in hospitals. It is composed of six items, grouped into two factors, corresponding to the dimensions of internal and external intention to leave. Item rating is based on a 7-point Likert-type scale (1, totally unlikely; 7, totally certain), in which high scores indicated a greater intention to leave. Considering the psychometric properties, the Intention to Leave Scale showed high reliability, with Cronbach’s alpha ranging from 0.92 (internal intention to leave) to 0.94 (external intention to leave). 

The Portuguese version of the Organizational Commitment Questionnaire by Meyer and Allen [27] includes 19 items, rated on a 7-point Likert-type scale (1, totally disagree; 7 Totally agree), with the higher scores corresponding to a higher organizational commitment. The questionnaire has a three-dimensional structure (affective, continuance, and normative organizational commitment dimensions). Data analysis used the simplified structure adapted to the Portuguese nursing context by Neves, Graveto, Rodrigues, Marôco, and Parreira [28]. The questionnaire had an internal consistency of α_affective organizational commitment_ = 0.82; α_continuance organizational commitment_ = 0.65; and α_normative organizational commitment_ = 0.81 [28].

The Practice Environment Scale of the Nursing Work Index by Lake [29] translated into the Portuguese context by Amaral, Ferreira, and Lake [30], comprised 31 items, rated on a 4-point Likert-type scale (1, strongly agree; 4, strongly disagree), then inverted. The higher scores correspond to favorable practice environments. The analysis used the version with a global factor, which was adapted to the Portuguese culture context by Neves, Parreira, Graveto, Rodrigues, and Marôco [31]. The scale had good psychometric properties, in the Portuguese context, with an internal consistency of α = 0.91.

Information related to the professional situation included variables, such as education level, professional title of specialist nurse, the exercise of specialized functions, contractual relationship (individual employment contract, employment contract in public function or other), service, and length of professional experience.

The indicators for staffing were provided by the nurse managers/management services. The necessary staffing was estimated according to the Regulatory Directive No. 1/2006 (Circular Normativa No. 1/2006) in use in the Portuguese NHS [32]. The estimation considered indicators, such as capacity, occupancy rate, nursing hours per patient day (according to the nursing patient classification system), working period, and hours worked by nurses. The safe staffing was estimated by Regulation no. 533/2014 (Regulamento No. 533/2014), of 2 January (proposed by the Portuguese Nurses Order) [33]. This uses the same calculation formula, but updates the nursing hours per patient day and introduces a weighting factor associated with the support provided by operational assistants. The adequacy of staffing was set up by the percentage deviation between actual staffing and necessary/safe staffing, based on the formula [(actual staffing—necessary (or safe) staffing): actual staffing × 100]. 

### 2.3. Data Analysis

Descriptive (measures of central tendency, dispersion, and frequency) and inferential analysis were performed using the Statistical Package for the Social Sciences software (version 22.0, SPSS An IBM Company, Chicago, IL, U.S.A.). Kolmogorov–Smirnov test results revealed no normal distributions of all the analyzed variables, so non-parametric Mann–Whitney (two samples) and Kruskal–Wallis (more than two samples) tests were carried out, followed by the multiple comparisons of mean ranks with Fisher’s LSD test [34]. The Pearson’s correlation coefficient was used to analyze the intensity and direction of the association between continuous (structure) variables. The statistical significance was set at *p* ≤ 0.05.

### 2.4. Ethical Considerations

This study is a part of major research on safe staffing and quality of nursing care, approved by the Board Directors and Ethics Committees of the hospitals, as well as the Ethics Committee of the Faculty of Medicine of the University of Coimbra (Proc. CE-100/2014). The ethical principles for the research were complied with, including the attainment of written informed consent, anonymity, and confidentiality. 

## 3. Results

The sample consists of 850 nurses (response rate: 46.10%), mostly female, between 22 and 59 years old (81.86%), as shown in Table 1. There is a prevalence of graduate nurses (89.05%), and 27% of them also have a specialty degree. Of these specialists, only 33% work as specialists. The time frame relating to professional experience varies between a few months and 38 years. The individual employment contract is the most common contractual relationship (59.70%), followed by an employment contract in public functions (38.94%). A residual number of professionals have other precarious contracts (1.36%), being hired from temporary employment companies. Approximately half of the nurses work in internal medicine services (50.82%), and the rest in general surgery (26.24%) and orthopedics (22.94%).

In consideration of the analysis of intention to leave, 34.50% of nurses manifest an internal intention to leave, that is, a desire to change services, and only 8.71% manifest an external intention to leave (a desire to change hospital). According to Table 2, on average, the nurses’ intention to leave their service is not clear (M = 3.62; SD = 1.86), and intention to leave the hospital even less (M = 2.21; SD = 1.32). In regard to organizational commitment, affective commitment is high (M = 4.09; SD = 1.25), and so is continuance commitment (M = 4.62; SD = 1.45), with normative commitment being lower (M = 3.26; SD = 1.20). The work environment is perceived by nurses as unfavorable (M = 2.39; SD = 0.47), with an inadequate availability of nursing staff. A mean nurse shortage of −5.38% was found when comparing actual staffing with necessary staffing laid out in Regulatory Directive no. 1/2006, a value that amounts to −36.73% based on safe staffing (Regulation No. 533/2014).

Table 2 shows the significant negative correlations between the nurses’ internal and external intention to leave, structure variables (nurse staffing, work environment, and organizational commitment), and age (*p* ≤ 0.05). Thus, older age and favorable organizational structural conditions tend to decrease the intention to leave.

Additionally, internal intention to leave is positively associated with external intention to leave, that is, the greater the intention to change service, the greater the intention to leave the hospital (r = 0.416; *p* < 0.001). 

There is a statistically significant positive association between affective organizational commitment (r = 0.478; *p* < 0.001) and normative organizational commitment (r = 0.452; *p* < 0.001) in relation to the work environment. Affective organizational commitment also presents a significant positive association with staffing deviation from both regulations, with continuance organizational commitment being positively associated with an adequacy of staffing based only on Regulatory Directive No. 1/2006 (*p* ≤ 0.05). In regard to the variables identified, a better work environment and a lower percentage deviation between actual staffing and estimated staffing mean a higher commitment of nurses to the organization.

In regard to the analysis of intention to leave and organizational commitment according to the contractual relationship, Mann–Whitney test results (Table 3) show that internal and external intention to leave is significantly different among nurses, according to the contractual relationship (*p* ≤ 0.001). Nurses with individual employment contracts manifest a greater intention to leave than those with an employment contract in public functions.

Continuance organizational commitment is also significantly different (*p* ≤ 0.001), according to the contractual relationship. Nurses with individual employment contracts present higher mean continuance organizational commitment scores, compared to those with an employment contract in public functions. However, affective and normative organizational commitment is not significantly different among nurses, regarding the contractual relationship. 

Given the current importance of the contractual relationship for career advancement opportunities, the relation between the professional development opportunities dimension of the Practice Environment Scale of the Nursing Work Index and external and internal intention to leave (r = −0.169; r = −0.306) was also assessed, which proved to be statistically significant (*p* < 0.001).

In regard to the hypothesis that the titles of specialist and working as a specialist do not determine intention to leave and organizational commitment, the Mann–Whitney test results show no significant differences in internal intention to leave between the specialist nurses and non-specialist nurses. The values at the threshold of statistical significance were observed (*p* = 0.050) in external intention to leave. On the other hand, Table 4 shows that nurses who do not work as specialists have significantly higher levels of internal intention to leave, compared to those who do work as specialists (*p* < 0.001).

In relation to organizational commitment, significantly higher continuance and normative organizational commitment levels were found in non-specialized nurses compared to nurse specialists (*p* < 0.05). Additionally, nurse specialists who do not work as such, present significantly higher continuance organizational commitment levels, compared to those who work as specialists (*p* = 0.012).

Finally, the Kruskal–Wallis test results showed no significant differences in the organizational commitment of nurses, according to the specialty of the unit in which they work (*p* > 0.05). However, the unit’s specialty shows a statistically significant effect of internal (*p* ≤ 0.001) and external (*p* = 0.002) intention to leave. 

**Figure 1:**
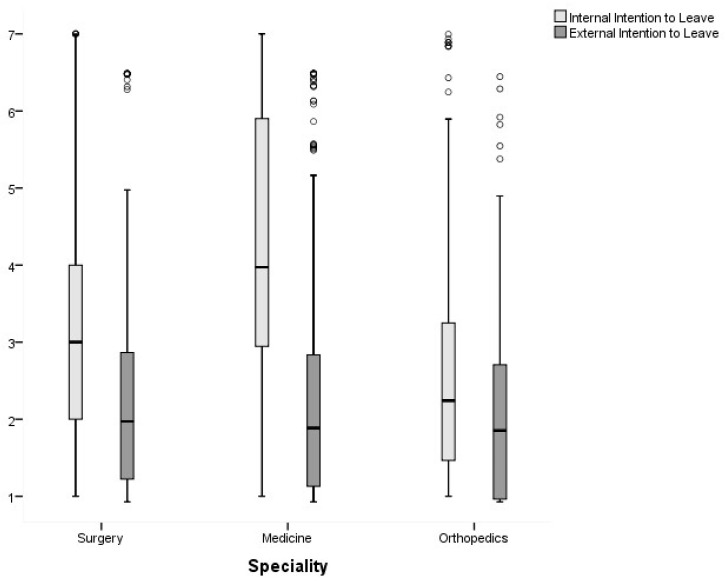
Distribution of nurses’ internal and external intent to leave, according to the unit’s specialty.

The multiple comparison of the mean ranks shows significant differences in internal intention to leave (*p* ≤ 0.001) between all specialties, with nurses who work in internal medicine services manifesting a greater intention to leave. Regarding the external intention to leave, significant differences were found between orthopedics and the other specialties (*p* ≤ 0.05). There were no differences (*p* = 0.409) between surgery and medicine, with orthopedic nurses presenting the lowest levels of external intention to leave, as shown in Figure 1. 

## 4. Discussion

The global shortage of nurses increased the interest in analyzing the intention to leave as a strong predictor of turnover. The turnover and intention to leave phenomena are very complex and have so many determinants and implications in the quality of care and sustainability of health systems [35,36]. This study indicates a moderate rate of nurses’ intention to leave the service (internal intention to leave: 34.50%). However, the nurses’ intention to leave the hospitals analyzed in the present study is low (external intention to leave: 8.71%), compared to a previous study in Portuguese hospitals, for which the response rate was higher (42.5%) [24]. In the international context, the rate of the nurses’ organizational turnover intention is also globally higher. For example, previous studies identified 14% in the U.S.A.x, 15% in Germany, 21.9% in Italy, 49% in Greece, and 52% in Turkey [14]. 

From the analysis of organizational commitment, the results of this study show that affective and continuance organizational commitment is high, with normative organizational commitment being lower than the midpoint. These results are congruent with a study developed in a Portuguese hospital, which also identifies lower values of normative commitment [37]. The continuance dimension of commitment is highly dependent on employment opportunities. Thus, high continuance organizational commitment may result from the widespread perception of the scarcity of alternatives in other healthcare organizations. The same may also motivate the nurses’ reduced external intention to leave. A study of Thai nurses identified a significant direct and indirect effect of employment opportunities on the intention to leave [38]. Thus, nurses remain in the organization because they “need” to, given the reduced supply of alternatives. The austerity measures in the Portuguese NHS, particularly on the nurses working conditions, may also affect dissatisfaction and instability, which may induce a feeling of weak obligation and moral duty towards the organization [5,37].

In opposition to other studies that show high levels of continuity commitment in older and more experienced nurses [5], the results expose a significant inverse relationship between the variables of age and continuance organizational commitment. The younger nurses tend to be more willing to remain at work. These results can be justified by the influence of job opportunities (which are reduced in the Portuguese context) on this type of commitment. 

Additionally, the intention to leave tends to decrease with age. Previous studies pointed to younger nurses as a risk group for turnover [4,20,39]. The inverse association between the nurses’ age and intention to leave reflects the need to improve the working conditions in younger age groups. The results are consistent with previous studies, alerting to the importance of retaining young professionals. Strategic intervention in this area may mitigate its future effect on the profession and health systems, as the intention to leave may evolve to abandoning the profession and aggravate the already evident shortage of nurses [4,20,39].

Intention to leave can be triggered by individual factors, but also by adverse external/organizational factors, such as the work environment. Sometimes, the intention to leave, as a psychological response, progresses to the cognition culminating in the leave (turnover) [40]. 

In this study, external and internal intention to leave was inversely associated with the structure factors analyzed in the present study. Higher organizational commitment, more favorable work environments and better adequacy of staffing contribute to a lower intention to leave. On the other hand, there is a tendency for greater internal and external intention to leave when the shortage of nurses is higher, and work environments are unfavorable to practice. 

The negative relationship between commitment and turnover was identified in other studies, including in different activity sectors. Organizational commitment is often considered to be a predictor of intention to leave, having a mediating effect on the relationship with other structure variables [6,19,38,41]. These results highlight the need to invest in developing nurses’ organizational commitment to reduce the intention to leave and, subsequently, turnover. 

Nevertheless, it is important to point out that the effect of continuance organizational commitment on professional turnover is sometimes only noticeable in the short term. It may even become counterproductive because employees’ perceptions of the costs and benefits of leaving the organization can lead to a minimum effort to maintain the contractual relationship. In turn, productivity decreases due to the absence of affective commitment and job satisfaction [42].

Previous national and international studies also highlighted this relationship between the work environment and intention to leave. Consistently with the results obtained, adequate work environments, thus being favorable to practice, should minimize intention to leave [1,8,12,24,43] and, consequently, the time/cost associated with integration processes and the risk of loss in quality of care provided during the replacement periods. Thus, ensuring favorable work environments is crucial to mitigate intentions to leave and promote nurse retention. Despite the significant relationship of the work environment with the intention to leave, being identified as a determinant of it, a Thai study suggests that it has an indirect influence, mediated by job satisfaction and commitment [6,38].

The association between nurse staffing and nurses’ intention to leave was discussed in previous studies. Previous studies emphasize the relationship between the staffing of nurses and exit intention. The contexts in which patient–nurse ratios are higher, show greater intention to leave [4,13,44]. Work overload can lead to increased tension, stress, and burnout. High levels of burnout reduce organizational commitment and, simultaneously, increase intention to leave and turnover [4,7,43]. Thus, is essential to invest in the policies about the nurse workforce [1,38]. This has a double positive effect by improving working conditions to reduce the intention to leave.

Work overload may also be the cause for the difference in the levels of intention to leave manifested by nurses of different specialty services, because of the increased complexity of and need for nursing care. Professionals in internal medicine services present a greater intention to leave the unit, followed by those in general surgery and orthopedics. Orthopedic nurses manifest a significantly lower external intention to leave than those from medicine and surgery units. This likely reflects the increase in nursing care hours per patient day, over the years, with an increase of 65% in internal medicine, 34% in general surgery, and 33% in orthopedics (Regulation No. 533/2014) [33]. However, this increase in needs did not result in adequate nursing staffing in the Portuguese NHS, where estimated staffing is still based on the care hours per patient day of 2005 (Regulatory Directive No. 1/2016) [32]. Therefore, in internal medicine services, a greater work overload is associated with a shortage of nurses (greater increase in care hours per patient day), hence a greater internal intention to leave. On the other hand, from a global point of view, orthopedics reported the smallest increase in care hours per patient day, which may lead to lower internal and external intention to leave in nurses from this specialty.

From the results of the study, it can be observed that nurses who have more precarious contractual employment relationships have higher levels of intention to leave. This finding can be due to the perception of opportunities for professional development and valorization that, according to the results, is negatively related to intention to leave. For individual employment contracts, the contractual relationship is consolidated in the Labor Code (equivalent to the private sector) and does not contemplate the possibility of career progression associated with the employment contract in public functions. Discontent and demotivation, due to non-recognition of personal investment in professional development and a scarcity of opportunities to perform differentiated functions, contribute to specialized nurses’ desire to change work context, seeking better working conditions. Previous studies found that the opportunities for professional development significantly influence the nurses’ intention to leave, highlighting the need to promote professional development, progression, and valorization [24,43,45].

Furthermore, there is greater continuance organizational commitment among younger professionals with an individual employment contract, balancing its effect on the intention to leave. This finding is probably also due to the scarcity of job offers in nursing in Portugal. Despite the evident shortage of nurses in the Portuguese NHS, job offers have decreased because of austerity measures, namely, human resource cost cuts. In addition to the increased weekly work hours (from 35 h to 40 h/week), the need for hiring decreased, and additional restrictions were also imposed, including in the replacement of professionals leaving the Portuguese NHS [22].

The results also show that the professionals with a greater specialized skill differentiation tend to show a greater desire to move to another organization (values at the threshold of the statistical evidence). Nurses who do not provide specialized care, manifest a significantly high internal intention to leave. The absence of opportunities for professional development and valorization is, possibly, also at the base of this relationship. A national study already highlighted this association [24], while, at the international level, nurses with higher qualifications (graduates vs. nongraduates) tend to manifest greater intention to leave [4,14].

These results are an important contribution to knowing the organizational context in public Portuguese hospitals. In this context, it is crucial to review the human resource management policies in the Portuguese NHS, particularly about the equity of professionals’ contractual terms. Indeed, the discrepancy in the opportunities for professional development and valorization seems to translate into higher levels of external intention to leave. The results allow for the support of a strategic intervention, adapted to specific contextual needs, to minimize the effect of intention to leave on quality of care.

Nurse managers play a key role at this level. They should motivate nurses through job enrichment, including the sustained creation of demanding and stimulating conditions, while ensuring autonomy and job-based feedback, as proposed by Hackman and Oldham [46,47]. This is particularly necessary for younger professionals, whose contractual relationship to the Portuguese NHS is an individual employment contract, with no possibility of career progression, as well as for those who do not provide specialized care according to their professional qualifications. Thus, it is vital to invest in the recognition and valorization of professional performance and development. Additionally, improving unfavorable and short-resourced work settings is key to increasing professional retention and subsequently promoting health outcomes.

A recent study reinforces this need by identifying personal achievement and job satisfaction as attractive factors for nurses. It also considers that an investment in work environment improvement and adequate staffing in terms of numbers and skills is vital to respond to growing needs in health care [7].

This measure is also consistent with the association between leadership and intention to leave, as shown by the results of previous studies, which recognize the influence of work environments and burnout. Effective and proper leadership, promoting favorable environments to practice, and facilitating the minimization of burnout among workers, may contribute to avoiding turnover and improving quality of care [48].

Furthermore, an OECD report [49] stresses that the sustainability of the Portuguese NHS may suffer because of its incapability of retaining professionals, associated with an aging population. The same report highlights the motivation of employees, especially nurses, as a challenge for the Portuguese NHS to reduce intention to leave. On the other hand, better working conditions in the private sector and abroad drive professionals out, reflected by the increase, in recent years, in nurse emigration in Portugal [49].

The use of new leadership strategies in health, namely, through integrated responsibility centers that act as intermediate organic management structures with functional autonomy, could facilitate the improvement of human resource management effectiveness. Additionally, the models of family health unit organization, at the level of primary health care, while allowing for self-forming teams, lead to a higher collaboration and complementarity, which may result in more committed employees. Integrated responsibility centers and family health units, united toward the accountability, autonomy, and engagement of employees, also increase productivity and job satisfaction. Supported by ad hoc, decentralized models, they empower professionals and create favorable conditions for their staying in organizations. Recognizing and rewarding individual and collective performance, for the promotion of professional and organizational development, can minimize intention to leave and facilitate professional retention. It can also foster the continuous improvement of results and quality, thus increasing value in health systems.

A recent proposal for a “Model of Organizational Commitment Applied to Health Management Systems”, identifies the need for transformational leadership that promotes organizational commitment and well-being [18].

The limitations of the study are identified, so we should be cautious in interpreting the results. The design and the sampling method (by convenience) in the selection of the hospitals and nurses make it impossible to generalize the results and establish causal relationships between the variables. In addition, the use of self-reported questionnaires can cause bias.

Further studies, with probabilistic sampling, and including other care settings, namely, primary and long-term care, are essential to understand these phenomena. This is particularly relevant in the current context, given the impact of the COVID-19 pandemic on health systems, being crucial to understanding its influence on nurses’ organizational commitments and intentions to leave, and to support the nurse workforce policies and strategies.

## 5. Conclusions

Healthcare organizations are continuously challenged to avoid waste, to promote health gains, and the sustainability of health systems. This is particularly relevant considering the growing health needs of the population, associated with the shortage of nurses.

Despite a low intention to leave among nurses, this study highlights the relation between intention to leave and individual and organizational factors, namely, age, organizational commitment, academic qualifications, contractual relationship, opportunities for professional development, work environment, adequacy of staffing, as well as the unit’s specialty.

The results show the urgent need for intervention in less favorable contexts, with inadequate staffing to respond to care needs, especially in internal medicine inpatient units.

The results are a relevant contribution to decision-making in health policies and human resource management. Evidence-based intervention strategies are proposed for the promotion of employee commitment, highlighting the importance of opportunities for professional valorization and development to reduce the intention to leave.

## Figures and Tables

**Table 1:** Participant’s characteristics.

Participant’s Characteristics	Mean	Standard Deviation	*n*	%
Age (years)	36.11	7.97		
Gender				
Male			152	18.14
Female			686	81.86
Education level				
Bachelor’s degree (3-year degree)			10	1.19
Bachelor’s degree (4-year degree)			748	89.05
Master’s degree			80	9.52
Doctoral degree			2	0.24
Title of specialist nurse				
Yes			222	27.07
Specialization area				
Rehabilitation nursing			122	59.22
Medical-surgical nursing			57	27.67
Mental health and psychiatric nursing			10	4.86
Community nursing			9	4.37
Maternal health and obstetrics nursing			8	3.88
Working as a specialist nurse				
Yes			70	33.98
No			136	66.02
Length of professional experience (years)	12.99	7.80		
Employment contract				
Individual employment contract			483	59.70
Public service employment contract			315	38.94
Other			11	1.36
Service specialty				
General surgery			223	26.24
Internal medicine			432	50.82
Orthopedic			195	22.94

**Table 2:** Descriptive and correlational analysis of nurses’ intention to leave, organizational commitment, work environment, adequacy of staffing, and age (*n* = 850).

	Mean	Standard Deviation	IITLes	EITL	AOC	COC	NOC	NPWE	Dev.06 ^†^	Dev.14 ^†^	Age ^‡^
IITL	3.62	1.86	1								
EITL	2.21	1.32	0.416 **	1							
AOC	4.09	1.25	−0.406 **	−0.462 **	1						
COC	4.62	1.45	−0.087 *	−0.213 **	0.114 **	1					
NOC	3.26	1.20	−0.342 **	−0.360 **	0.768 **	0.264 **	1				
NPWE	2.39	0.47		−0.239 **	0.478 **	0.000	0.452 **	1			
Dev. 06 ^†^	−5.38	17.50	−0.084 *	−0.084 *	0.121 **	0.097 **	0.065	0.022	1		
Dev.14 ^†^	−36.73	27.71	−0.216 **	−0.092 **	0.086 *	0.061	0.046	0.017	0.859 **	1	
Age ^‡^	36.11	7.97	−0.172 **	−0.096 **	0.027	−0.189 **	−0.058	0.006	0.015	0.142 **	1

IITL, internal intention to leave; EITL, external intention to leave; AOC, affective organizational commitment; COC, continuance organizational commitment; NOC, normative organizational commitment; NPWE, nursing practice work environment; Dev. 06, percentage deviation between actual staffing and estimated staffing based on Regulatory Directive No. 1/2006; and Dev. 14, percentage deviation between actual staffing and estimated staffing based on the Regulation No. 533/2014. ^†^ *n* = 835; ^‡^ *n* = 827. * Significant correlation at *p* ≤ 0.05; ** Significant correlation at *p* ≤ 0.001.

**Table 3:** Results of the Mann–Whitney test on the intention to leave and organizational commitment according to the contractual relationship.

	Contract	*n*	Mean	Standard Deviation	U	*p*
Internal intention to leave	IEC ^1^	483	3.95	1.90	56,423.50	<0.001
	ECPF ^2^	315	3.12	1.70		
External intention to leave	IEC	483	2.35	1.42	65,313.50	0.001
	ECPF	315	2.02	1.15		
Affective organizational commitment	IEC	483	4.02	1.21	71,861.50	0.186
	ECPF	315	4.15	1.28		
Continuance organizational commitment	IEC	483	4.78	1.36	64,792.50	<0.001
	ECPF	315	4.37	1.57		
Normative organizational commitment	IEC	483	3.25	1.12	72,565.50	0.271
	ECPF	315	3.20	1.25		

^1^ IEC: individual employment contract; ^2^ ECPF: employment contract in public functions.

**Table 4:** Results of the Mann–Whitney test on the intention to leave and organizational commitment, according to nurses’ specializations.

	Nurses’ Specializations	*n*	Mean	Standard Deviation	U	*p*
Internal intention to leave	Specialist	222	3.72	1.95	63,740	0.381
	Non-specialist	598	3.57	1.82		
	Works as specialist	70	2.87	1.49	3041	<0.001
	Does not work as a specialist	136	4.15	2.01		
External intention to leave	Specialist	222	2.33	1.32	60,482	0.050
	Non-specialist	598	2.16	1.31		
	Works as specialist	70	2.25	1.25	4429	0.414
	Does not work as a specialist	136	2.38	1.37		
Affective organizational commitment	Specialist	222	4.07	1.22	65,017	0.652
	Non-specialist	598	4.10	1.26		
	Works as specialist	70	4.14	1.13	4713	0.908
	Does not work as a specialist	136	4.05	1.24		
Continuance organizational commitment	Specialist	222	4.33	1.56	56,810	0.001
	Non-specialist	598	4.74	1.39		
	Works as specialist	70	3.88	1.63	3741	0.012
	Does not work as a specialist	136	4.47	1.50		
Normative organizational commitment	Specialist	222	3.09	1.12	58,077	0.006
	Non-specialist	598	3.32	1.21		
	Works as specialist	70	3.16	1.18	4754	0.988
	Does not work as a specialist	136	3.06	1.07		

## Data Availability

The data presented in this study are available on request from the corresponding author. The data are not publicly available due to restrictions to ensure privacy and anonymity.
